# Causality relationship between 91 inflammatory factors and 5 intestinal diseases: A two-sample bidirectional Mendelian randomized study

**DOI:** 10.1097/MD.0000000000045735

**Published:** 2025-11-14

**Authors:** Xiaokui Yuan, Tong Wang

**Affiliations:** a Department of Pharmacy, The Fourth People’s Hospital of Chengdu, The Clinical Hospital of Chengdu Brain Science Institute, University of Electronic Science and Technology of China, Chengdu, China; b Department of Clinical Laboratory, The Fourth People’s Hospital of Chengdu, The Clinical Hospital of Chengdu Brain Science Institute, University of Electronic Science and Technology of China, Chengdu, China.

**Keywords:** causality relationship, inflammatory factors, intestinal diseases, Mendelian randomization

## Abstract

Emerging evidence from recent pathological investigations has demonstrated that chronic inflammation plays a pivotal role in the pathogenesis of intestinal diseases, including inflammatory bowel disease (IBD), ulcerative colitis (UC), Crohn disease (CD), colorectal adenocarcinoma (CAC), and colorectal cancer (CRC). However, the precise regulatory mechanisms of inflammatory cytokines remain incompletely elucidated, and the causal relationships between inflammatory responses and intestinal diseases require further validation. This study employed a two-sample Mendelian randomization (MR) approach to comprehensively evaluate potential causal associations between 91 circulating inflammatory factors and these 5 intestinal diseases. MR analysis revealed a significant causal relationship between the levels of inflammatory factors C-C motif chemokine 19 and CD40L receptor and the risk of IBD. Furthermore, CD, UC and CRC respectively showed potential causal relationships with inflammatory factors C-C motif chemokine 20, Programmed cell death 1 ligand 1 and interleukin-5. No inflammatory factor showed a causal relationship with CAC. Reverse MR Analysis indicated that the progression of CRC could significantly regulate the expression level of the inflammatory factor Macrophage colony-stimulating factor 1. Unlike most previous studies that merely focused on the association between a certain inflammatory factor and intestinal diseases, this study was the first to systematically identify the relationships between 91 inflammatory factors and intestinal diseases based on the MR method. These results not only deepen our understanding of the inflammatory regulatory mechanism of intestinal diseases, but also provide a theoretical basis for the development of future clinical early diagnosis and targeted treatment strategies.

## 1. Introduction

Inflammatory bowel disease (IBD), comprising its principal subtypes ulcerative colitis (UC) and Crohn disease (CD), along with inflammation-associated intestinal malignancies including colorectal adenocarcinoma (CAC) and colorectal cancer (CRC), represent a substantial global burden of gastrointestinal pathology.^[1–3]^ Contemporary epidemiological surveillance demonstrates a marked escalation in IBD incidence worldwide over the past 2 decades, with prevalence rates increasing by over 30% and annual incidence in developed nations reaching 24.3 per 100,000 population.^[1]^ Concurrently, CRC accounted for more than 1.9 million new cases globally in 2020, ranking as the third most prevalent malignancy.^[2]^ While recent advances in immunomodulatory therapies (e.g., anti-TNF-α monoclonal antibodies) and targeted treatments (e.g., PD-1 inhibitors) have significantly improved clinical outcomes,^[4,5]^ approximately 40% of patients still experience therapeutic failure^[4]^ underscoring critical gaps in our understanding of disease pathogenesis.

Accumulating evidence confirms that chronic inflammatory responses secondary to intestinal barrier dysfunction constitute a fundamental pathological mechanism underlying disease progression. Preclinical investigations have demonstrated that aberrant activation of the IL-23/Th17 axis induces IBD-like enteritis in experimental models, while sustained TNF-α-mediated NF-κB signaling pathway activation is mechanistically linked to CRC pathogenesis.^[6,7]^ However, the clinical translation of these findings presents considerable challenges: anti-IL-17A monoclonal antibody therapy may paradoxically exacerbate inflammation in CD patients, while anti-TNF agents have failed to demonstrate anticipated prophylactic efficacy against CRC.^[8]^ This translational impasse highlights inherent limitations of conventional research paradigms-observational studies cannot reliably distinguish causal effects of inflammatory mediators from epiphenomena, while animal models inadequately recapitulate the complex gene-environment interactions characteristic of human disease.^[9]^

As central regulators of the immune microenvironment, circulating inflammatory factors have been extensively investigated for their associations with intestinal pathologies.^[10,11]^ Genome-wide association studies (GWAS) have identified polymorphisms in IL12B and IL23R loci among IBD susceptibility variants, providing compelling genetic evidence for the pivotal role of the IL-12/IL-23 pathway.^[12]^ Nevertheless, observational study designs remain susceptible to residual confounding factors (e.g., gut microbiota composition, unmeasured environmental exposures) and cannot exclude reverse causation. Consequently, more robust methodological approaches are urgently required to elucidate causal relationships between inflammatory factor dynamics and distinct intestinal disorders.

Mendelian randomization (MR) methodology offers a novel solution to this scientific challenge.^[13,14]^ This approach employs genetic variants as instrumental variables (IVs), with 3 principal advantages: the random allocation of genetic variants during gametogenesis effectively circumvents conventional confounding; genotype–phenotype associations are inherently protected from reverse causation by disease status; the analysis using the aggregated data of GWAS shows an improvement in efficiency and repeatability. These characteristics establish MR as an optimal tool for investigating exposure-outcome causality.

Although previous studies have utilized magnetic resonance imaging (MRI) to explore the association between specific inflammatory factors (such as C-reactive protein) and colorectal cancer (CRC),^[15]^ most of these studies have focused on a single regulatory factor or a limited disease spectrum^,[16]^ failing to provide a more comprehensive reflection of the connection between inflammatory factors and the disease. Furthermore, the existing research has mainly focused on common inflammatory factors, while neglecting the potential roles and clinical values of rare inflammatory factors in intestinal diseases. Therefore, using a more comprehensive dataset of inflammatory factors is of great significance for screening potential disease markers and therapeutic targets. Furthermore, given the clear pathological continuity between inflammatory bowel diseases (IBD, CD, UC) and intestinal tumors (CAC, CRC), as well as the significant role of inflammation in the development of intestinal diseases, conducting in-depth research on the causal relationships between various inflammatory factors and various intestinal diseases holds great scientific value.^[17]^ Accordingly, this study pioneers a large-scale bidirectional two-sample MR analysis to systematically evaluate causal associations between 91 inflammatory factors and 5 intestinal diseases. The findings may not only yield potential biomarkers for early disease detection but also provide novel theoretical frameworks for understanding disease etiology and pathogenesis.

## 2. Materials and methods

### 2.1. Data sources

**Figure 1.**
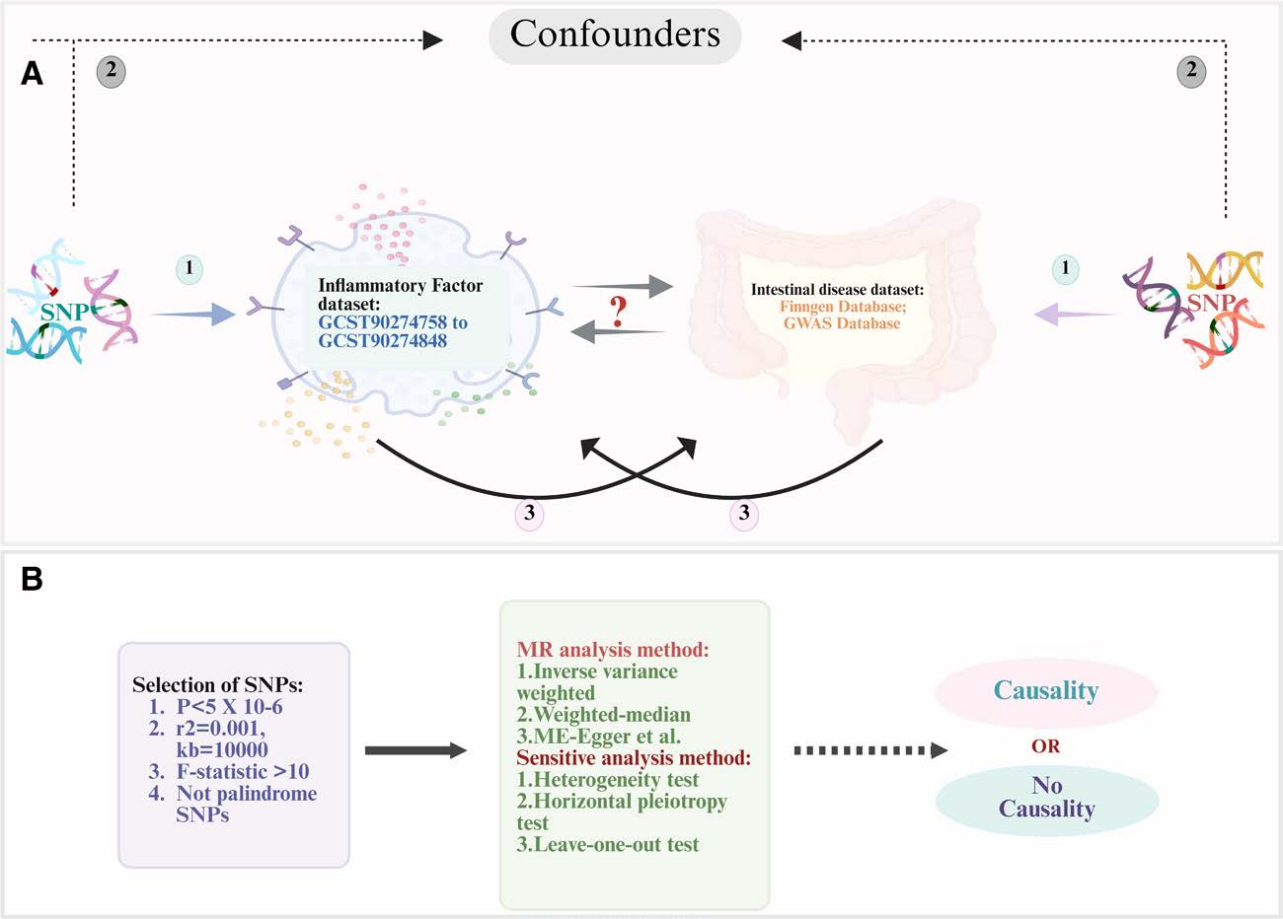
(A) Three assumptions in this MR study: ① Relevance: SNPs should be robustly associated with the exposure. ② Independence: SNPs are not related to any confounding factor that can influence exposure or outcome. ③ Exclusion restriction: SNPs only influence the outcome via the exposure. (B) Workflow of this MR study. The first time: 91 inflammatory factors were used as exposure factors, and 5 intestinal diseases were used as outcome for MR analysis. The second time: 5 intestinal diseases were used as exposure factors, and 91 inflammatory factors were used as outcome for MR analysis. MR = Mendelian randomization, SNP = single nucleotide polymorphism.

Fig. 1 illustrates the overall study design flowchart. All data were derived from published GWAS summary statistics. Specifically, the GWAS datasets for 91 circulating inflammatory factors (accession numbers: GCST90274758 to GCST90274848) were obtained from a genome-wide association analysis of protein quantitative trait loci (pQTLs) for 91 inflammation-related proteins in plasma samples from 14,824 participants of European ancestry.^[18]^ Summary statistics for the 5 intestinal diseases (IBD, UC, CD, CAC and CRC) were acquired from the FinnGen database and GWAS database (details provided in Table S1, Supplemental Digital Content, https://links.lww.com/MD/Q693). As this study utilized only publicly available datasets, neither patient consent nor additional ethical approval was required.

### 2.2. Study design

The study incorporated 2 complementary MR analytical approaches: forward MR analysis: evaluating potential causal associations between 91 inflammatory factors (exposures) and 5 intestinal diseases (outcomes); reverse MR analysis: assessing potential causal effects of 5 intestinal diseases (exposures) on 91 inflammatory factors (outcomes) (Fig. 1).^[18–23]^

### 2.3. Selection of IVs

Single nucleotide polymorphisms (SNPs) were selected as IVs using a genome-wide significance threshold (*P* < 5 × 10^−8^). To ensure independence among selected SNPs, we implemented linkage disequilibrium (LD)-based clumping with stringent parameters (*r*^2^ < 0.001, window size = 10,000 kb).^[24]^ The strength of each IV was quantified using the *F*-statistic, with *F* > 10 indicating sufficient instrument strength to minimize weak instrument bias.^[25]^ These selection criteria were consistently applied in both forward and reverse MR analyses. It is noteworthy that the dataset of intestinal diseases from the FinnGen database was used for the initial analysis, and the dataset of intestinal diseases from the GWAS database was used for the secondary validation.

### 2.4. Statistical analysis

Five complementary MR methods were employed to assess causal associations: inverse-variance weighted (IVW), weighted median, MR-Egger regression, weighted mode and simple mode. The IVW method provided the most efficient and consistent causal estimates when all SNPs satisfied valid IV assumptions, while MR-Egger regression offered more robust estimates in the presence of invalid instruments. Primary results were derived from IVW analyses, with other methods serving to verify robustness and consistency. Potential horizontal pleiotropy was evaluated using the MR-Egger intercept test (*P* < .05 considered significant), while heterogeneity among genetic variant estimates was assessed using Cochran *Q* statistic. All MR analyses and data processing were performed using RStudio software (version 4.1.2, packages: “Two Sample MR,” “forestplot,” and “MRPRESSO”). The code can be found in the supplementary materials (Supporting materials-The R code used in this research, Supplemental Digital Content, https://links.lww.com/MD/Q593).

## 3. Results

### 3.1. Characteristics of IVs and exposures

In strict accordance with our pre-established selection criteria, we identified SNPs significantly associated with 91 circulating inflammatory factors from published GWAS to serve as IVs. Similarly, disease-associated SNPs for the 5 intestinal disorders (IBD, CD, UC, CAC, and CRC) were extracted from the FinnGen database and GWAS database. Comprehensive baseline characteristics of both exposure (inflammatory factors) and outcome (intestinal diseases) variables, including sample sizes and detailed phenotype definitions, are systematically presented in Table S1 (Supplemental Digital Content, https://links.lww.com/MD/Q693). Given the considerable analytical breadth encompassing 5 distinct intestinal pathologies and 91 inflammatory mediators, we have focused our primary reporting on statistically significant causal associations (*P* < .05) derived from our MR analyses. The complete set of positive findings from both forward and reverse MR analyses is comprehensively documented in Tables S2–S11 (Supplemental Digital Content, https://links.lww.com/MD/Q693). All incorporated SNPs demonstrated *F*-statistics exceeding our stringent threshold of 10, thereby effectively eliminating concerns regarding weak instrument bias and ensuring robust causal inference.^[25]^ Furthermore, sensitivity analyses including MR-Egger intercept tests (all *P* > .05) and Cochran *Q* statistics provided no evidence of substantial horizontal pleiotropy or heterogeneity that might compromise the validity of our causal estimates.

### 3.2. The causative impact of 91 inflammatory factors on 5 intestinal diseases

**Figure 2.**
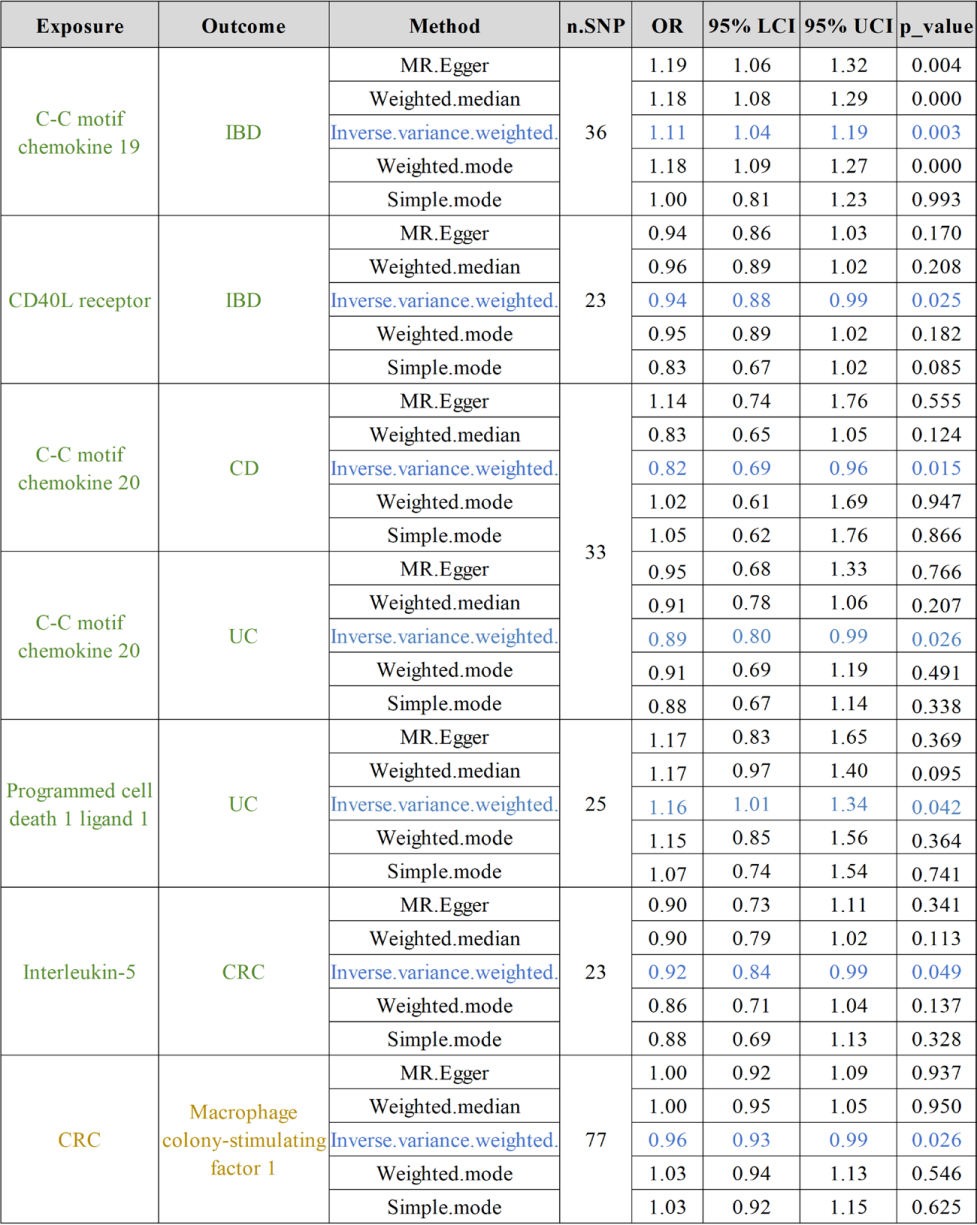
The pooled OR results between inflammatory factors and intestinal diseases (IBD, UC, CD, CAC, CRC) in the forward MR analysis and reverse MR analysis. CAC = colorectal adenocarcinoma, CD = Crohn disease, CRC = colorectal cancer, IBD = inflammatory bowel disease, MR = Mendelian randomization, UC = ulcerative colitis.

The IVW analysis results examining the causal relationships between 91 inflammatory factors (exposures) and 5 intestinal diseases (outcomes) are presented in Figure 2.

Results from the IVW analysis demonstrated that genetically predicted levels of the inflammatory factors C-C motif chemokine 19 (CCL19) (OR = 1.11, 95% CI = 1.04–1.19, *P* = .003) was significantly positively associated with an elevated risk of IBD. Conversely, CD40L receptor (CD40L or CD40) (OR = 0.94, 95% CI = 0.88–0.99, *P* = .025) was observed to be positively associated with IBD risk.

The IVW analysis revealed that genetically determined concentrations of the inflammatory chemokines C-C motif chemokine 20 (CCL20) (OR = 1.20, 95% CI = 1.05–1.38, *P* = .009) was positively associated with the risk of CD.

The IVW analysis demonstrated that genetically predicted concentrations of C-C motif chemokine 20 (CCL20) (OR = 0.89, 95% CI = 0.80–0.99, *P* = .026) exhibited a significant inverse correlation with the risk of UC. Conversely, Programmed cell death 1 ligand 1 (PD-L1) (OR = 1.16, 95% CI = 1.01–1.34, *P* = .042) was observed to be positively associated with UC risk.

The results of the IVW analysis demonstrated that no inflammatory factors exhibited significant inverse associations with the risk of CAC.

The findings derived from the IVW analysis indicated that the level of the inflammatory factor interleukin-5 (IL-5) (OR = 0.92, 95% CI = 0.84–0.99, *P* = .049) may be negatively correlated with the risk of colorectal cancer. The statistical *P*-value is only .049, which was <.05, and the statistical effect was not completely significant. In addition, apart from the IVW method, the results of the OR values obtained by the other 4 analysis methods (MR-Egger, weighted median, weighted mode, and simple mode) were consistent in direction with the OR values obtained through IVW, which to some extent indicates that IL-5 may be related to CRC. The results of the heterogeneity test and the multiplicity test were both >.05, indicating that the results do not have consistency and multiplicity (Fig. 2).

**Figure 3.**
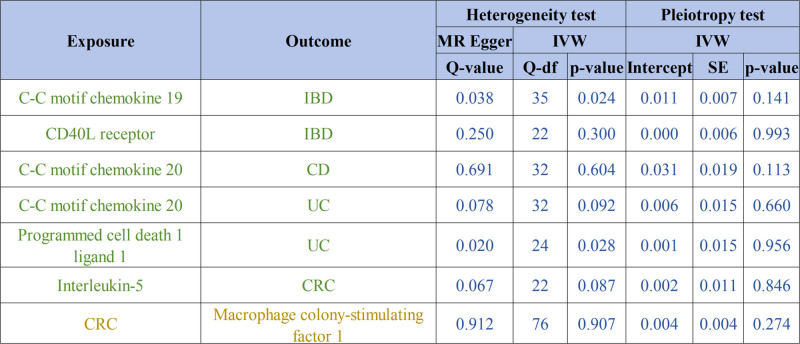
The heterogeneity and horizontal pleiotropy results of the inflammatory factors and intestinal diseases (IBD, UC, CD, CAC, CRC) in the forward MR analysis and reverse MR analysis. CAC = colorectal adenocarcinoma, CD = Crohn disease, CRC = colorectal cancer, df = degree of freedom, IBD = inflammatory bowel disease, MR = Mendelian randomization, *Q* = heterogeneity statistic *Q*, SE = standard error, UC = ulcerative colitis.

**Figure 4.**
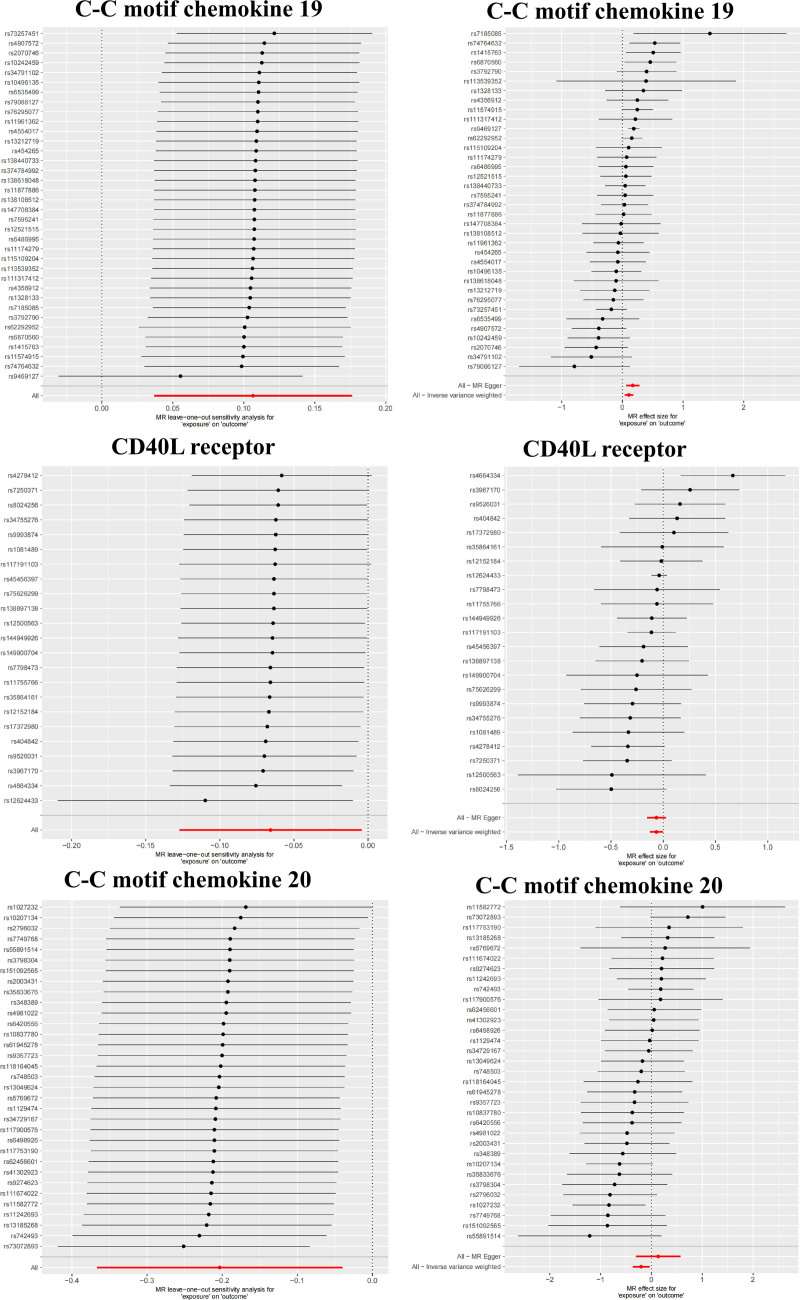
Mendelian randomization analysis. Leave-one-out plot and Forest plot of inflammation factors to intestinal diseases (C-C motif chemokine 19 to IBD, CD40L receptor to IBD and C-C motif chemokine 20 to CD). CD = Crohn disease, CD40 = CD40L receptor, IBD = inflammatory bowel disease.

**Figure 5.**
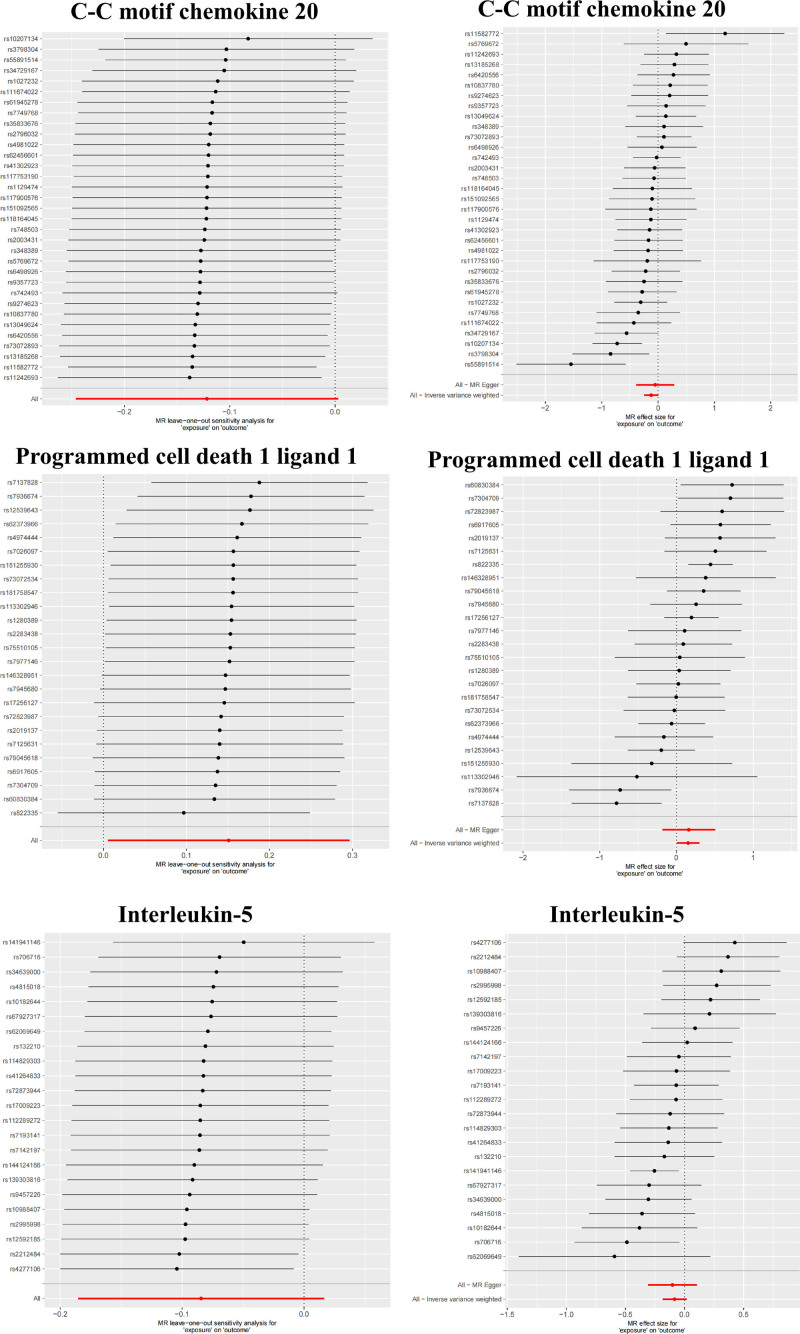
Mendelian randomization analysis. Leave-one-out plot and Forest plot of inflammation factors to intestinal diseases (C-C motif chemokine 20 to UC, Programmed cell death 1 ligand 1 to UC and Interleukin-5 to CRC). CRC = colorectal cancer, UC = ulcerative colitis.

The results of the heterogeneity test indicated that, within the positive MR analyses, significant heterogeneity was not observed for the majority of inflammatory factors. Conversely, the pleiotropy test demonstrated that none of the other factors exhibited significant horizontal pleiotropy (Fig. 3). Moreover, the effects of the causal associations were further corroborated by scatter plots, forest plots, and sensitivity analyses, which collectively reinforced the robustness and stability of these findings (Figs. 4 and 5; Figs. S1 and S2, Supplemental Digital Content, https://links.lww.com/MD/Q593).

### 3.3. Causal effects of intestinal diseases on 91 inflammatory factors

To further elucidate the potential influence of intestinal diseases on inflammatory factors, a reverse MR analysis was conducted. Specifically, 5 distinct intestinal diseases were used as exposures, while 91 inflammatory factors served as outcomes. The results of this analysis were depicted in Figure 2.

Analysis predicated on the IVW method indicated that CRC was associated with diminished levels of Macrophage colony-stimulating factor 1. The IVW analysis revealed that CRC may precipitate a reduction in the levels of Macrophage colony-stimulating factor 1 (M-CSF) (OR = 0.96, 95% CI = 0.93–0.99, *P* = .026). Moreover, findings from alternative MR methods – namely, MR-Egger regression, weighted median, weighted mode, and simple mode analyses – were concordant with those derived from the IVW methodology (Fig. 2).

**Figure 6.**
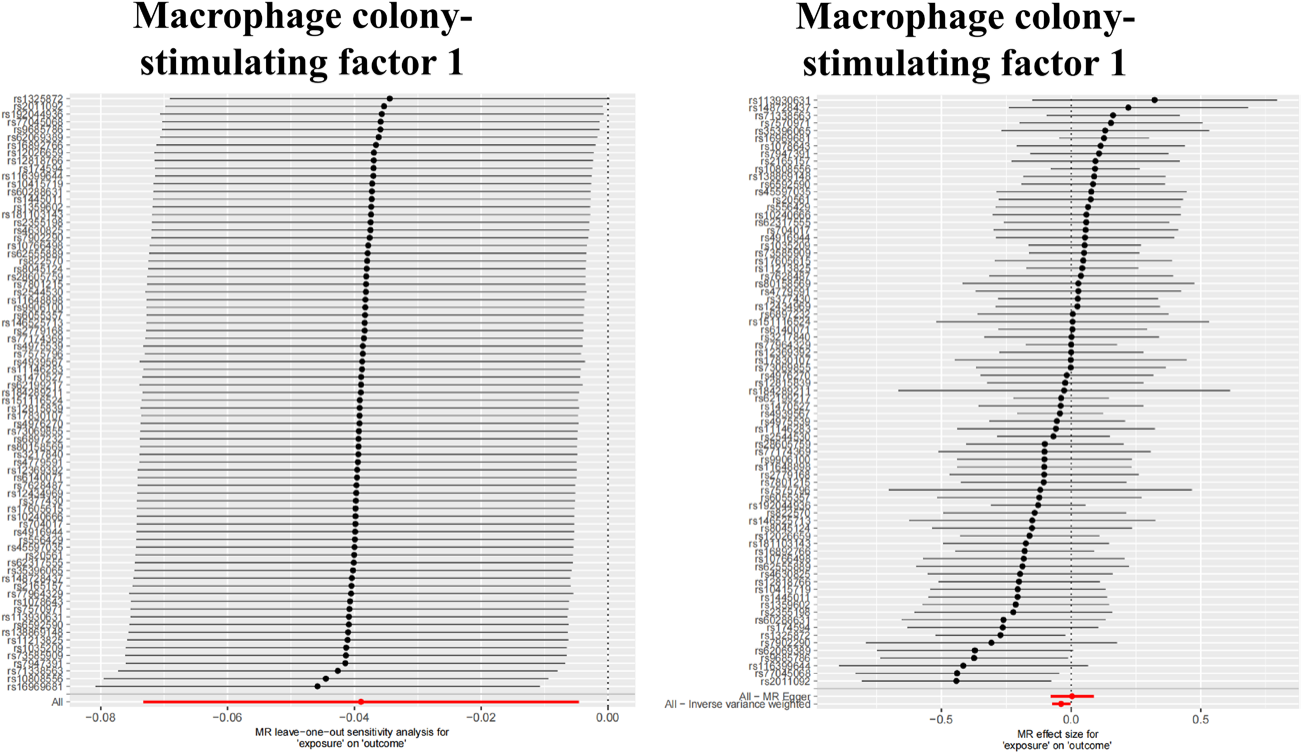
Mendelian randomization analysis. Leave-one-out plot and Forest plot of CRC to Macrophage colony-stimulating factor 1. CRC = colorectal cancer.

Heterogeneity testing revealed no substantial heterogeneity in the reverse MR analyses (Fig. 2). Pleiotropy assessment demonstrated no significant horizontal pleiotropy for the majority of findings (Fig. 2). Scatter plots, forest plots, and sensitivity analyses further corroborated the robustness of these observed causal associations (Fig. 6; Fig. S3, Supplemental Digital Content, https://links.lww.com/MD/Q593).

In contradistinction to conventional observational studies, this research leveraged MR analysis to furnish novel evidence for appraising the causal relationship between circulating inflammatory mediator levels and the risk of intestinal disorders. Nevertheless, the current study is subject to certain limitations. Firstly, this MR investigation provides solely statistical causal evidence pertaining to the association between circulating inflammatory mediators and intestinal diseases; consequently, the underlying biological mechanisms remain to be elucidated. Secondly, while sensitivity analyses did not detect significant pleiotropy, the potential influence of pleiotropy on the MR results cannot be entirely discounted. Finally, the genetic data utilized in the present study were predominantly derived from European populations; therefore, whether these findings are generalizable to individuals of other ethnic/geographical origins necessitates further investigation.

## 4. Discussion

Inflammatory factors play an indispensable role in the pathogenesis and progression of IBD, encompassing CD and UC. These molecules intricately regulate immune responses, intestinal barrier integrity, and cellular proliferation, thereby orchestrating disease advancement.^[10]^ Moreover, a markedly increased risk of intestinal neoplasia, including CAC and CRC, has been documented in IBD patients, particularly in those with disease duration exceeding 10 years.^[26]^ Although the pivotal roles of specific inflammatory mediators in these intestinal disorders have been preliminarily elucidated, the causal relationships between numerous other cytokines and IBD warrant further investigation. Using novel data and methodological approaches, this bidirectional MR study offers genetic insights into the potential causal links between inflammatory factors and 5 distinct gastrointestinal diseases.

C-C chemokines, defined by the presence of an N-terminal C-C structural domain, play critical roles in immune system cells (e.g., dendritic cells, macrophages, and NK cells) and tumor biology. In cancer cells, C-C chemokines primarily mediate biological functions including proliferation, anti-apoptosis, drug resistance, migration, and invasion.^[27]^ Studies demonstrate that serum CCL19 levels are significantly elevated in patients with rheumatoid arthritis and markedly reduced posttreatment, suggesting its potential as a biomarker for disease activity and therapeutic response.^[28]^ However, reports on CCL19 in IBD remain extremely limited, and its precise role in IBD pathogenesis remains unclear. Our findings are the first to establish a causal relationship between CCL19 and IBD, indicating that elevated CCL19 levels may promote IBD progression and could serve as a potential therapeutic target for IBD.

The CD40 ligand receptor (CD40) is predominantly expressed on the surface of immune cells such as B cells and dendritic cells. Upon binding to CD40L (CD154) on activated T cells, it drives antigen presentation and B cell activation.^[29]^ The CD40/CD40L axis functions as a pivotal signaling transduction pathway between immune cells.^[30]^ Our findings reveal a significant inverse correlation between CD40L expression levels and IBD, suggesting that these mediators may exert protective functions via suppressing hyperactive immune responses. As a co-stimulatory molecule, CD40L is essential for T cell activation; although prior studies predominantly emphasize its pro-inflammatory roles, the observed reduction in its levels in IBD patients may reflect impaired regulatory T cell activity or dysregulation of immune tolerance.^[31]^ The results of this study confirm the causal relationship between CD40 and IBD, further supporting the CD40/CD40L signaling pathway as a novel therapeutic target for IBD.

CCL20, a member of the CC chemokine family, exerts its effects via binding to the chemokine receptor CCR6.^[32]^ During active IBD, both the chemokine ligand CCL20 and its receptor CCR6 are upregulated in colonic biopsy samples.^[33]^ In CD, abnormally elevated mucosal CCL20 drives Th17 cell infiltration, exacerbating mucosal damage.^[34]^ Here, our results further confirm the causal relationship between CCL19 and IBD. Its immunosuppressive properties in autoimmune diseases and tumors provide a critical rationale for developing targeted therapies. Additionally, in human UC-associated cancer, upregulated CCL20 expression in the colonic epithelium increases susceptibility to colorectal cancer.^[35]^ Bioinformatic analyses also identify CCL20 as a key gene in UC pathogenesis, a finding consistent with our observations.^[36]^

Programmed death (PD)-1 and its ligands PD-L1 and PD-L2 are negative co-stimulatory molecules that regulate T cell motility and the formation of immune synapses between T cells and antigen-presenting cells.^[37]^ In UC patients, the relative expression levels of PD-L1 and PD-L2 genes are significantly higher than in controls.^[38]^ Our results also demonstrate a positive correlation between PD-L1 levels and UC progression. Given that dysregulation of the PD-1/PD-L1 axis is a hallmark of immune dysfunction in UC, future studies should focus on both forms of PD-1/PD-L1 signaling molecules to better elucidate UC pathogenesis and identify potential therapeutic targets.^[39]^

The interplay between chronic inflammation and colorectal cancer (CRC) involves complex processes, including cellular activation within the tumor microenvironment and immune cell infiltration. Inflammatory factors produced by immune cells play pivotal roles in tumorigenesis. Cytokines such as IL-5 promote tumor development and metastasis while suppressing apoptosis by activating the signal transducer and activator of transcription 3 (STAT3) transcription factor.^[40]^ IL-5 is primarily secreted by Th2 cells and mast cells. In CRC, tumor cells may induce local inflammatory responses, triggering immune cells to secrete IL-5, thereby enhancing the recruitment and activation of eosinophils. Furthermore, M-CSF directly augments the invasive capacity of colorectal cancer cells and accelerates metastatic progression by binding to its receptor and activating downstream signaling pathways (e.g., PI3K/AKT).^[41]^ Serum M-CSF levels are significantly elevated in CRC patients compared to healthy individuals, suggesting its potential as a noninvasive prognostic biomarker.^[42,43]^ The findings of this study further elucidate the causal relationships of M-CSF with CRC, providing novel directions for clinical early detection and improved patient prognosis. Furthermore, the causal relationship between IL-5 and CRC as shown in the results of this study was not yet fully established. More data or further research is needed to further verify this.

This MR study employed publicly accessible datasets derived from the FinnGen database and GWAS database, with its substantial sample size and utilization of robust IVs underpinning the robustness and credibility of the findings. Unlike traditional observational research, this investigation introduces novel evidence supporting the causal relationship between inflammatory factor levels and the risk of intestinal diseases through MR analysis.^[9]^ Nonetheless, several limitations warrant consideration. First, whilst the current MR framework can provide statistical evidence of a causal link between circulating inflammatory factors and IBD, the underlying mechanistic pathways remain to be elucidated through further research. Secondly, although the sensitivity analyses performed did not reveal any significant pleiotropy among the SNPs, the potential influence of pleiotropic effects on the MR estimates cannot be entirely discounted. Lastly, the genetic data utilized in this study predominantly originates from individuals of European descent, raising questions regarding the generalisability of these findings to populations from other ethnic backgrounds or geographical regions. Future research should aim to validate these results across diverse cohorts, investigate the specific biological mechanisms by which inflammatory factors influence disease progression, and expand the demographic scope to enhance the representative of the findings.

## 5. Conclusion

In summary, our MR analysis has provided genetic evidence supporting the causal effects of multiple inflammatory factors – such as CCL19, CD40L, CCL20, PD-L1, IL-5, and M-CSF – on 5 distinct intestinal diseases (IBD, UC, CD, CAC, and CRC). These inflammatory factors hold promise as potential biomarkers for the early detection of the aforementioned intestinal disorders and may also serve as prospective therapeutic targets for targeted interventions. Nonetheless, the precise mechanistic pathways underpinning how each of these inflammatory mediators influences the pathogenesis of these conditions remain to be elucidated through further investigative efforts.

## Acknowledgments

The authors thanks the support of the Fourth People’s Hospital of Chengdu.

## Author contributions

**Data curation:** Xiaokui Yuan.

**Formal analysis:** Xiaokui Yuan.

**Supervision:** Tong Wang.

**Validation:** Xiaokui Yuan, Tong Wang.

**Visualization:** Xiaokui Yuan, Tong Wang.

**Writing – original draft:** Xiaokui Yuan.

**Writing – review & editing:** Tong Wang.

## Supplementary Material




