# Supplementary material for: Causality relationship between 91 inflammatory factors and 5 intestinal diseases: A two-sample bidirectional Mendelian randomized study
Source: Medicine (Baltimore). 2025 Nov 14;104(46):e45735. doi: 10.1097/MD.0000000000045735 (PMC12622616; doi:10.1097/MD.0000000000045735)
Supplement: Supplementary file 2 [file medi-104-e45735-s002.pdf]

```
library(TwoSampleMR)
library(data.table)
library(tidyverse)
library(readxl)
library(writexl)
library(mr.raps)
library(ieugwasr)
library(dplyr)
library(MendelianRandomization)
library(purrr)
library(magrittr)
library(tidyr)
library(openxlsx)

rm(list = ls())
gc()
setwd("D:/MR-91YZYZ")

FileNames <- list.files(paste0(getwd()), pattern = ".tsv")
exp_dat_ids <- FileNames


exps <- sapply(FileNames, function(x) {
  str_remove(x, "\\.tsv$") %>%
    str_replace_all("_\\.tsv", "_")
})

outcome_files <- list.files("outcome/", pattern = "\\.gz$", full.names = TRUE)

dir.create(path = "mendelian test", showWarnings = FALSE)


get_f<-function(dat,F_value=10){
  log<-is.na(dat$eaf.exposure)
  log<-unique(log)
  if(length(log)==1)
  {if(log==TRUE){
    print("数据不包含 eaf，无法计算 F 统计量")
    return(dat)}
  }
  if(is.null(dat$beta.exposure[1])==T || is.na(dat$beta.exposure[1])==T){print("数据不包含 beta，
无法计算 F 统计量")
    return(dat)}
```

```
  if(is.null(dat$se.exposure[1])==T || is.na(dat$se.exposure[1])==T){print("数据不包含 se，无法计算 F 统计量")
    return(dat)}
  if(is.null(dat$samplesize.exposure[1])==T  ||  is.na(dat$samplesize.exposure[1])==T){print("数据不包含 samplesize(样本量)，无法计算 F 统计量")
    return(dat)}


  if("FALSE"%in%log && is.null(dat$beta.exposure[1])==F && is.na(dat$beta.exposure[1])==F && is.null(dat$se.exposure[1])==F && is.na(dat$se.exposure[1])==F && is.null(dat$samplesize.exposure[1])==F && is.na(dat$samplesize.exposure[1])==F){
R2<-(2*(1-dat$eaf.exposure)*dat$eaf.exposure*(dat$beta.exposure^2))/((2*(1-dat$eaf.exposure)*dat$eaf.exposure*(dat$beta.exposure^2))+(2*(1-dat$eaf.exposure)*dat$eaf.exposure*(dat$se.exposure^2)*dat$samplesize.exposure))
    F<- (dat$samplesize.exposure-2)*R2/(1-R2)
    dat$R2<-R2
    dat$F<-F
    dat<-subset(dat,F>F_value)
    return(dat)
  }
}



steiger_test <- function(dat) {
  dat$r.exposure <- get_r_from_bsen(b = dat$beta.exposure,
                                     dat$se.exposure,
                                     dat$samplesize.exposure)
  dat$r.outcome <- get_r_from_bsen(b = dat$beta.outcome,
                                    dat$se.outcome,
                                    dat$samplesize.outcome)
  res_steiger <- mr_steiger(
    p_exp = dat$pval.exposure,
    p_out = dat$pval.outcome,
    n_exp = dat$samplesize.exposure,
    n_out = dat$samplesize.outcome,
    r_exp = dat$r.exposure,
    r_out = dat$r.outcome
  )
  res_steiger <- directionality_test(dat)

  return(res_steiger)
}
```

```
results_binary <- function(N, alpha, R2xz, K, OR, epower) {
    threschi <- qchisq(1 - alpha, 1) # threshold chi(1) scale
    f.value <- 1 + N * R2xz / (1 - R2xz)

    if (is.na(epower)) {

        b_MR <- K * ( OR/ (1 + K * (OR - 1)) -1)

        v_MR <- (K * (1-K) - b_MR^2) / (N*R2xz)
        NCP <- b_MR^2 / v_MR

        # 2-sided test
        power <- 1 - pchisq(threschi, 1, NCP)
        data.frame(Parameter = c("Power", "NCP", "F-statistic"), Value = c(power, NCP, f.value),
Description = c("", "Non-Centrality-Parameter", "The strength of the instrument"))


    } else {

        # Calculation of sample size given power
        z1 <- qnorm(1 - alpha / 2)
        z2 <- qnorm(epower)
        Z    <- (z1 + z2)^2

        b_01 <- K * ( OR/ (1 + K * (OR - 1)) -1)
        f <- K * (1-K) - b_01^2
        N1 <- Z * f / (b_01^2 * R2xz)
        N1 <- ceiling(N1)
        data.frame(Parameter = "Sample Size", Value = N1)

    }
}


#####MR-PRESSO######
# PRESSO
mr_Presso<-function(dat,num=10000){
    library(TwoSampleMR)
    library(MRPRESSO)
    library(dplyr)
    set.seed(123)
    try (mr_presso_res<-mr_presso(BetaOutcome ="beta.outcome", BetaExposure =
```

```
"beta.exposure", SdOutcome ="se.outcome", SdExposure = "se.exposure",
                                                OUTLIERtest = TRUE,DISTORTIONtest = TRUE, data = dat,
                                                SignifThreshold = 0.05,NbDistribution = num))
  return(mr_presso_res)

}
mr_presso_pval<-function(mr_presso_res){
  try ( mr_presso_main<-mr_presso_res$`Main MR results`)
  try ( mr_presso_main[3:5,]<-NA)
  return(mr_presso_main)
}


mr_presso_snp<-function(mr_presso_res,mr_presso_main,dat,type="list"){
  data_re<-list()
  if(type=="list"){
    for(i in 1:length(mr_presso_res)){
      res<-mr_presso_res[[i]]
      main<-mr_presso_main[[i]]
      data<-dat[[i]]
      try(if(is.na(main[2,6])==FALSE){
        outliers<-which(res$`Outlier Test`$Pvalue<0.05)
        data$mr_keep[outliers]<-FALSE
      })
      data_re[[i]]<-data
      names(data_re)[[i]]<-names(dat)[[i]]
    }
    return(data_re)
  }

  if(type=="data"){
    res<-mr_presso_res$`MR-PRESSO results`
    main<-mr_presso_main
    data<-dat
    try(if(is.na(main[2,6])==FALSE){
      outliers<-which(res$`Outlier Test`$Pvalue<0.05)
      data$mr_keep[outliers]<-FALSE
    })
    return(data)
  }
}
```

```
choose_MR <- function(dat = dat) {
    res_hete <- NULL
    if (nrow(dat) < 3) {
        res <- mr(dat, method_list = c("mr_ivw", "mr_wald_ratio"))
    } else {
        res_hete <- mr_heterogeneity(dat)
        if (res_hete$Q_pval[2] < 0.05) {
            res <- mr(dat, method_list = c(
                "mr_egger_regression", "mr_weighted_median", "mr_ivw_mre",
"mr_weighted_mode", "mr_simple_mode"
            ))
        } else {
            res <- mr(dat, method_list = c(
                "mr_egger_regression", "mr_weighted_median", "mr_ivw_fe", "mr_weighted_mode",
"mr_simple_mode"
            ))
        }
    }
    AAA <- list(res_hete, res)
    return(list(AAA))
}

for (outcome_file in outcome_files) {
    current_outcome <- try(fread(outcome_file, header = TRUE), silent = TRUE)
    head(current_outcome)
    current_outcome <- current_outcome[ ,-9]
    head(current_outcome)
    colnames(current_outcome) <- c("chr","pos", "effect_allele", "other_allele", "beta", "se", "eaf",
"pval", "SNP", "samplesize")
    head(current_outcome)


    outcome_name <- basename(outcome_file) %>%
        str_remove("\\.gz$") %>%
        str_remove("_\\.gz")




    for (qaq in 1:length(exp_dat_ids)) {
        exp_dat_id <- exp_dat_ids[qaq]
        exp <- exps[qaq]

        exp2 <- tools::file_path_sans_ext(exp)
```

```
    outcome_name2 <- tools::file_path_sans_ext(outcome_name)

    outcome_dir <- file.path("mendelian test", outcome_name2)
    dir.create(outcome_dir, showWarnings = FALSE)


    d3 <- try(fread(paste0(getwd(), "/", exp_dat_id), fill = TRUE), silent = TRUE)



    if (inherits(d3, "try-error") || nrow(d3) == 0) {
      message(paste("无法读取或空暴露文件:", exp_dat_id, "，跳过该暴露"))
      next
    }


    d3 <- subset(d3, pval < 1e-5)
    d3 <- d3[d3$SNP!="", ]

    if (nrow(d3) == 0) {
      message(paste("筛选后无有效数据:", exp_dat_id, "，跳过该暴露"))
      next
    }

    d3 <- as.data.frame(d3)
    d3 <- format_data(d3, type = "exposure")


    d4 <- tryCatch({
      ld_clump(
        dplyr::tibble(rsid = d3$SNP, pval = d3$pval.exposure, id = d3$id.exposure),
        clump_kb = 10000,
        clump_r2 = 0.001,
        pop = "EUR",
        plink_bin                                                                    =
"C:/Users/17911/Desktop/MR-shipin+daima/plink_win64_20241022/plink.exe",
        bfile = "C:/Users/17911/Desktop/MR-shipin+daima/EUR/EUR"
      )
    }, error = function(e) {
      message(paste("在 clump", exp, "后无有效 SNP，ld_clump  出现错误：", e$message, "，
跳过当前循环。"))
      return(NULL)
    })
```

```
if (is.null(d4)) {
    next
}

exp_data <- subset(d3, SNP %in% d4$rsid)

if (nrow(exp_data) == 0) {
    message(paste("无有效 SNP:", exp, "，跳过该暴露"))
    next
}


outcome_dat <- merge(exp_data, current_outcome, by.x = "SNP", by.y = "SNP", all.x = TRUE)


temp_file <- file.path(outcome_dir, paste0("temp_", exp2, "_", outcome_name2, ".csv"))
write.csv(outcome_dat, file = temp_file, row.names = FALSE)


out_data <- read_outcome_data(
    snps = exp_data$SNP,
    filename = temp_file,
    sep = ","
)
out_data <- format_data(outcome_dat, type = "outcome")

file.remove(temp_file)


out_data <- subset(out_data, pval.outcome > 5e-8)
dat <- harmonise_data(exposure_dat = exp_data, outcome_dat = out_data)

if (nrow(dat) == 0) {
    message(paste("无匹配 SNP:", exp2, "与", outcome_name2, "，跳过该组合"))
    next
}


dat <- subset(dat, mr_keep == TRUE)

if (nrow(dat) == 0) {
    message(paste("无有效 SNP:", exp2, "与", outcome_name2, "，跳过该组合"))
    next
}
```

```r
# dat$samplesize.outcome <- 411317
# dat$samplesize.exposure <- 7174


dat <- get_f(dat, F_value = 10)

dat <- subset(dat, mr_keep == TRUE)
res <- choose_MR(dat)


res_hete <- map(seq_along(res), ~ res[[.x]][[1]]) %>% do.call(rbind, .)

res1 <- generate_odds_ratios(res[[1]][[2]])
res1$estimate <- paste0(
    format(round(res1$or, 2), nsmall = 2), " (",
    format(round(res1$or_lci95, 2), nsmall = 2), "-",
    format(round(res1$or_uci95, 2), nsmall = 2), ")"
)


openxlsx::write.xlsx(dat, file.path(outcome_dir, paste0(exp2, "_", outcome_name2,
"-dat.xlsx")), rowNames = FALSE)
openxlsx::write.xlsx(res1, file.path(outcome_dir, paste0(exp2, "_", outcome_name2,
"-res.xlsx")))


res_steiger <- steiger_test(dat)


library(magrittr)
# Main result
res3 <- res1#[1:5,]
res3 <- res3[,-c(10:14)]
res4 <- tidyr::pivot_wider(
    res3,names_from ="method",names_vary = "slowest",
    values_from = c("b","se","pval","estimate") )

col_names <- colnames(res4)

new_col_names <- gsub("\\(.*\\)", "", col_names)

colnames(res4) <- new_col_names
```

```
res_steiger2 <- dplyr::select(res_steiger,
                              correct_causal_direction,steiger_pval)


res_ALL <- cbind(res4, res_steiger2)
res_ALL$F <- mean(dat$F, na.rm = TRUE)
res_ALL$R2 <- sum(dat$R2)


if (nrow(dat) <= 2) {
  write.csv(res_ALL, file.path(outcome_dir, paste0(exp2, "_", outcome_name2, "1.csv")),
row.names = FALSE)
} else {

  res_plei <- mr_pleiotropy_test(dat)
  res_leaveone <- mr_leaveoneout(dat)


  pdf(file.path(outcome_dir, paste0(exp2, "_", outcome_name2, "_scatter.pdf")))
  print(mr_scatter_plot(res[[1]][[2]], dat)[[1]])
  dev.off()

  pdf(file.path(outcome_dir, paste0(exp2, "_", outcome_name2, "_forest.pdf")))
  print(mr_forest_plot(mr_singlesnp(dat, all_method))[[1]])
  dev.off()

  pdf(file.path(outcome_dir, paste0(exp2, "_", outcome_name2, "_funnel.pdf")))
  print(mr_funnel_plot(mr_singlesnp(dat, all_method))[[1]])
  dev.off()

  pdf(file.path(outcome_dir, paste0(exp2, "_", outcome_name2, "_leave_one_out.pdf")))
  print(mr_leaveoneout_plot(res_leaveone))
  dev.off()


  res_hete2 <- pivot_wider(res_hete, names_from = method, values_from = c(Q, Q_df,
Q_pval)) %>%
    select(-id.exposure, -id.outcome, -outcome, -exposure) %>%
    select(4:6)

  res_plei2 <- res_plei %>% select(egger_intercept, se, pval)


  res_ALL <- cbind(res_ALL, res_hete2, res_plei2)
```

```
      write.csv(res_ALL, file.path(outcome_dir, paste0(exp2, "_", outcome_name2, ".csv")),
row.names = FALSE)
    }
  }


  rm(current_outcome)
  gc()

  csv_files <- list.files(file.path(outcome_dir), pattern = "\\.csv$", full.names = TRUE, recursive =
TRUE)
  combined_df <- map_df(csv_files, ~ {
    temp_df <- read.csv(.x, stringsAsFactors = FALSE)
    outcome_name <- basename(dirname(.x))
    temp_df$outcome <- outcome_name
    temp_df
  }) %>%
    mutate(fdr_Inverse.variance.weighted. = p.adjust(pval_Inverse.variance.weighted., method
= "fdr")) %>%
    relocate(fdr_Inverse.variance.weighted., .after = pval_Inverse.variance.weighted.)


  subset_df <- combined_df[combined_df$pval_Inverse.variance.weighted. < 0.05, ]
  subset_nsnp_df <- combined_df[combined_df$nsnp < 3, ]

  combined_df <- combined_df[combined_df$nsnp >= 3, ]


  subset_nsnp_df <- subset_nsnp_df[, colSums(is.na(subset_nsnp_df)) !=
nrow(subset_nsnp_df)]
  combined_df <- combined_df[, colSums(is.na(combined_df)) != nrow(combined_df)]

  wb <- createWorkbook()
  sheets <- list("sheet1" = combined_df, "sheet2" = subset_df, "sheet3" = subset_nsnp_df)
  walk2(names(sheets), sheets, ~ {
    addWorksheet(wb, .x)
    writeData(wb, .x, .y)
  })

  saveWorkbook(wb, file.path(outcome_dir, "results.xlsx"), overwrite = TRUE)
}
```

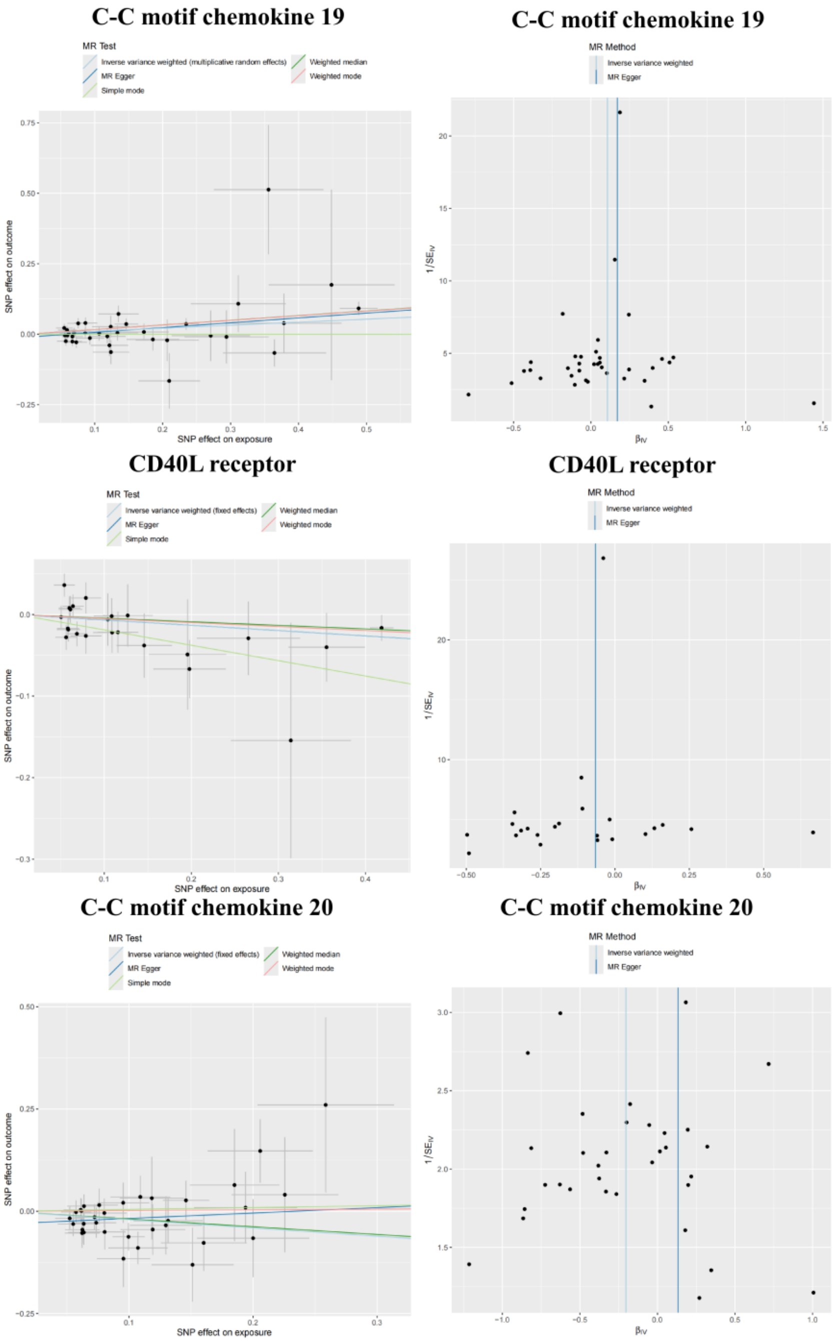

Fig S1. Mendelian randomization analysis. Scatter plot and funnel plot of inflammation factors to intestinal diseases (C-C motif chemokine 19 to IBD, CD40L receptor to IBD and C-C motif chemokine 20 to CD).

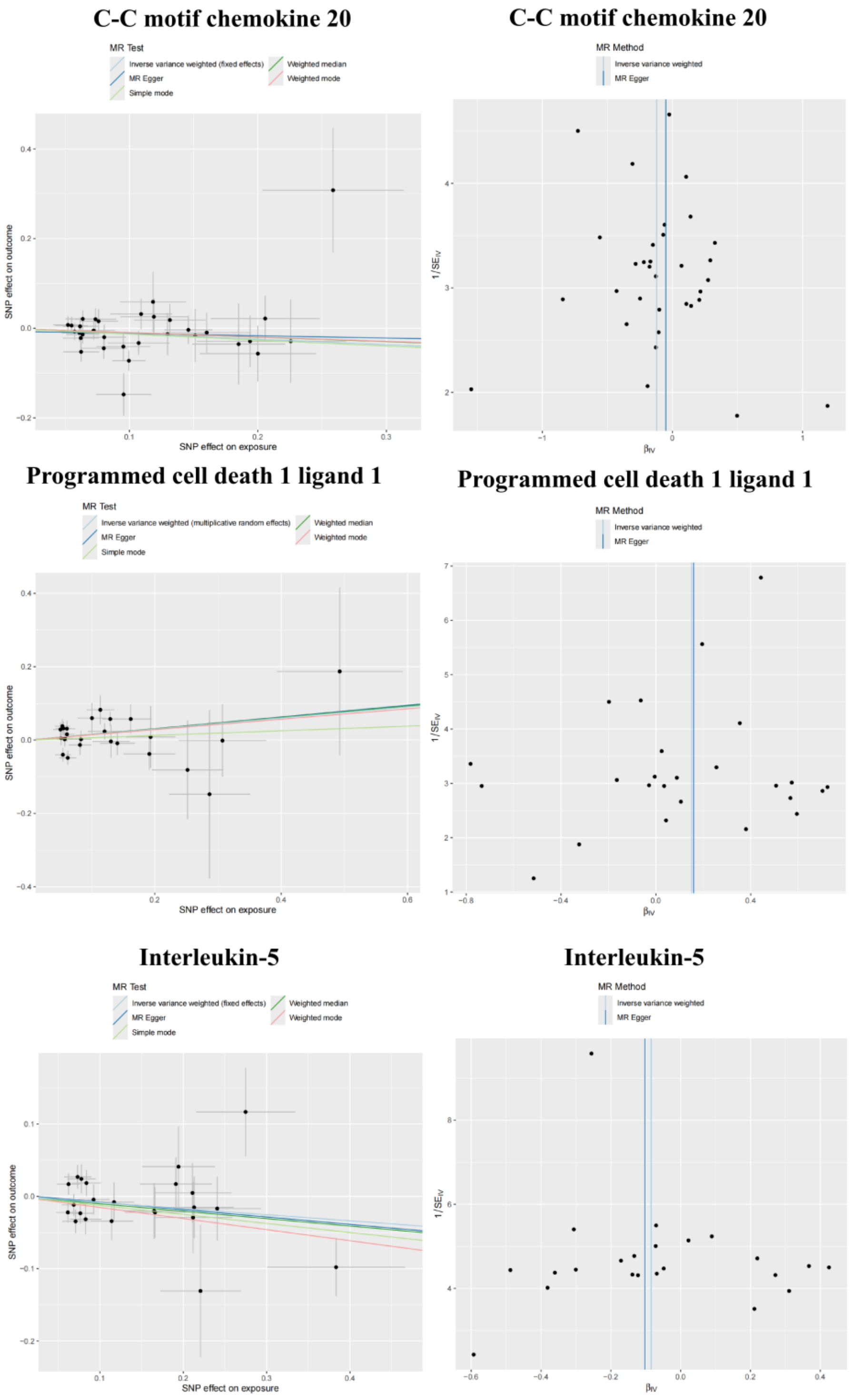

Fig S2. Mendelian randomization analysis. Scatter plot and funnel plot of inflammation factors to intestinal diseases (C-C motif chemokine 20 to UC, Programmed cell death 1 ligand 1 to UC and Interleukin-5 to CRC).

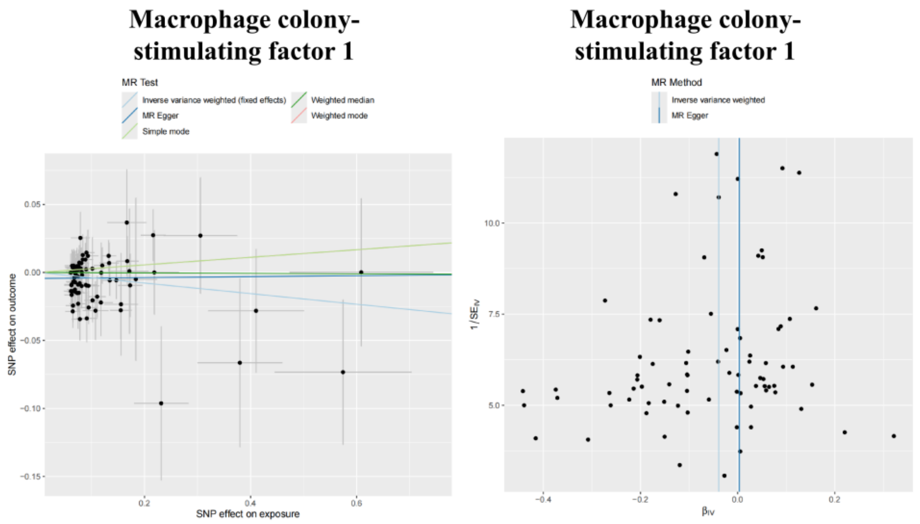

Fig S3. Mendelian randomization analysis. Scatter plot and funnel plot of CRC to Macrophage colony-stimulating factor 1.
